# Improving Knowledge, Engagement, and Self-Efficacy in the Creation of Healthy Home Environments for Mothers Using a Facebook Intervention (Design for Wellness): Randomized Controlled Trial

**DOI:** 10.2196/46640

**Published:** 2023-11-07

**Authors:** Tal Aperman-Itzhak, Isaac Prilleltensky, Laura Rosen

**Affiliations:** 1 Department of Health Promotion School of Public Health, Faculty of Medicine Tel Aviv University Tel Aviv Israel; 2 School of Education and Human Development University of Miami Miami, FL United States

**Keywords:** environmental home design, wellness, Facebook intervention, nudging, healthy living, social media, Israel

## Abstract

**Background:**

Designing the home environment can promote well-being. Social networks provide learning opportunities to improve health.

**Objective:**

This study aimed to develop and evaluate a Facebook intervention called Design for Wellness (DWELL). The program was created to improve knowledge, engagement, and self-efficacy in the creation of healthy home environments.

**Methods:**

A randomized controlled trial was conducted to assess the effects of the intervention program DWELL. Content was uploaded to the Facebook group and gave the participants practical solutions for how to design their home environment for wellness. The intervention addressed multiple components of health behaviors, such as healthy eating, physical activity, tobacco-free environment, hygiene, family conversations regarding wellness issues, and stress reduction. The main outcome was the participants’ overall score on the DWELL index, which we developed to assess the elements of our intervention: knowledge, awareness, engagement, and self-efficacy regarding home design for wellness. The intervention was conducted in Israel and lasted 6 weeks during the third wave of the COVID-19 pandemic. The primary analysis included a multivariable model to assess the DWELL score at the end of the study while controlling for baseline characteristics. The waitlist control group did not receive an intervention between the 2 administrations of the questionnaire.

**Results:**

In total, 643 participants began the program: 322 (50.1%) in the intervention group and 321 (49.9%) in the control group. Of the 643 participants, 476 (74%) completed the study. At the end of the study, there was a statistically significant benefit of the intervention as assessed using a one-way analysis of covariance: there was a mean difference of 8.631 (SD 1.408) points in the DWELL score in favor of the intervention group (intervention: mean 61.92, SD 14.30; control: mean 53.29, SD 16.374; *P*<.001). Qualitative feedback from participants in the intervention group strengthened the positive results as most of them found the group beneficial. The Facebook group was very active. Being more engaged in the group correlated with having a higher DWELL score, but this relationship was weak (*r*=0.37; *P*<.001). The mean significant difference of 26.281 (SD 19.24) points between the overall DWELL score and the overall engagement score indicated that participants who were not active in the group still followed the posts and benefited. We found no improvements in the secondary outcome regarding participants’ well-being. The COVID-19 lockdown may have prevented this.

**Conclusions:**

DWELL was found to be a beneficial intervention for improving perceptions of the design of home environments to foster wellness. Facebook was an effective platform to deliver this intervention. DWELL may become a prototype for other health promotion interventions.

**Trial Registration:**

ClinicalTrials.gov NCT03736525; https://clinicaltrials.gov/study/NCT03736525?term=DWELL&rank=1

## Introduction

### Background

Risk-related behaviors such as unhealthy diet, tobacco use, physical inactivity, and alcohol use increase the risk of noncommunicable diseases and premature death [1]. However, people still make poor decisions and harm their health. Decision-making processes are biased toward inertia; people usually go along with the status quo or the default option [2]. A *nudge* is a strategy for organizing the context in which people make decisions. Using nudges, it is possible to move people toward healthier choices. A nudge can alter people’s behavior in positive and predictable ways without curtailing their freedom of choice [2].

Nudges influence behaviors by changing the way choices are presented in the environment [3]. The environment has considerable power over people’s behavior. Environmental factors such as colors, sounds, smells, textures, temperature, and lighting may influence health behaviors related to food intake and choices. Availability and accessibility also appear to influence food and beverage consumption as people consume less food and drinks when the distance to them is longer [4]. Research also shows that people tend to eat more when the same food is separated into different bowls, the serving bowls are bigger, the food units are larger, the food is amorphous (making it harder to estimate portion size), the cup is wide (as opposed to tall), the food is colorful and varied, and temperatures are cold [4,5].

Although habits can be difficult to change and are influenced by cues in the environment [6], it is also possible to teach people how to create new habits and craft different cues [7]. One way to teach these skills is through social networks as these sites offer users opportunities for improving personal health. They are not only an information channel but also a communication channel. For example, in a qualitative content analysis of 38 college students, most participants referred to Facebook as the social networking site they used to keep informed about health and wellness [8]. In another web survey of 826 psychology students aged 18 to 25 years, a preference for Facebook was associated with heightened bonding social capital [9]. Facebook and other social networks facilitate informal learning because of their active role in members’ daily lives. Social networking sites support collaborative learning, enabling people to exchange ideas and share content through videos, photos, and links to external pages [10]. Facebook provides the largest array of functions, including text, photo sharing, and privacy settings, and it is used to build effective online communities [9,11]. Social media can also serve as a form of visual nudging to promote safe behaviors. In one study, universities that shared more mask-related visual content through social media such as Facebook and Instagram observed a significant decrease in COVID-19 positivity rates [11].

Online communities enable people to form social groups where they can give and receive social support and have fun [12]. In online communities, participants are characterized as having a shared sense of belonging and identifying with one another. Community identity is seen as one of the important determinants of an individual’s motivation to participate in web-based communities [10,13]. Perceived similarity between members of a group increases the cohesion among its members and enhances trust. Membership in an online community that is based on shared identity or perceived similarity, such as Facebook, is likely to foster goodwill and trust in its community members. Motherhood is the basis of a shared identity that is perceived to be trustworthy [14].

Online social interactions can increase social participation and psychological well-being [15]. Wellness or well-being is a positive state of affairs in different domains of life, including subjective (self-worth, autonomy, and belonging) and objective (sufficient economic resources, physical health, and access to healthy foods) states [16,17]. In this study, we used these 2 terms synonymously. Social networks such as Facebook support socialization in groups as they bring people together around shared interests and needs and, hence, facilitate community identity and participation [10].

### Objectives

This study aimed to develop and evaluate an online Facebook intervention named Design for Wellness (DWELL) to improve knowledge, engagement, and self-efficacy in the creation of healthy home environments. Home is one of the most important settings for promoting health and wellness [18]. According to the setting approach following the 1986 launch of the Ottawa Charter for Health Promotion, health is created and lived by people within the settings of everyday life [19]. Although nudging is a beneficial strategy for behavior change in the public health sector [20], it is a relatively new concept, and there is a need for more rigorous studies, including randomized controlled trials (RCTs) [6,21]. Hence, we used an RCT with quantitative outcomes to provide participants with an accessible and convenient online setting for active and group-oriented collaborative learning in an informal, personalized environment. An online intervention can be delivered easily to a wide range of populations. With a low budget, it is possible to maintain a Facebook group and keep its sustainability going for the long term. Currently, as far as we know, there is no successful Facebook group in Israel that serves as an online community encouraging participants to design their home environment for wellness. Furthermore, only a few health interventions on social media are based on experimental designs, and there are validity challenges associated with these interventions. In this regard, it is worth noting that health interventions on social media are in their early phases of development and more research is needed [22]. This study aimed to improve perceptions of participants regarding designing their home environment for wellness. The program was created to improve knowledge, engagement, and self-efficacy in the creation of healthy home environments. If the intervention is found to be beneficial, this group may become a useful tool in health promotion and lifestyle interventions and a prototype for similar interventions elsewhere.

## Methods

### Objectives, Design, and Development of the DWELL Intervention

The RCT of the DWELL intervention was the final step of a multiphase study aimed at developing, piloting, implementing, and evaluating an online Facebook intervention for designing the home environment for wellness in a population of mothers of young children.

#### Objectives

The intervention had five main objectives:

1. Primary objective: to improve the participants’ overall score on the DWELL index (as measured via knowledge and awareness, engagement, and self-efficacy regarding DWELL).
2. Secondary objective: to improve each of the components of the DWELL index separately (knowledge and awareness, engagement, and self-efficacy).
3. Secondary objective: to improve participants’ wellness (as measured using specific items of the I COPPE [Interpersonal, Community, Occupational, Physical, Psychological, Economic, and Overall Well-Being] scale and the 5-item World Health Organization Well-Being Index [WHO-5] instruments).
4. Secondary objective: to evaluate the associations between overall engagement score in the Facebook group and overall DWELL score.
5. Secondary objective: to evaluate the associations between levels of engagement with the Facebook group and specific items of the DWELL index.

#### Intervention

We created a new Facebook group for this program. The group served as an interactive platform, encouraging participants to design their environments, interact with one another, and engage in an online community for designing homes for wellness. The content was delivered in enjoyable and evidence-based ways. The participants could engage with wellness issues while chatting with members within the community, asking questions, uploading posts, sharing new ideas, and enjoying social support from the group. Involving participants is premised on the community participatory research approach, which suggests that outcomes are more successful when the study population participates in the design of the intervention [23].

All components of this study, including the intervention and evaluation, were online. The content was uploaded as posts to the Facebook group and gave the participants practical solutions on how to design their home environment for wellness in a reliable way based on scientific evidence. We defined “design the home environment” as the organization of the internal home surroundings, such as where things are placed, what we see first, and what is accessible to us and what is stored out of sight. Designing the home environment means modifying the cues within the environment, for example, redesigning the refrigerator so that healthy food items are at eye level, placing a plate of freshly cut vegetables on the dinner table every day, serving food on small plates, and using a sandglass to help children brush their teeth for 2 minutes. We developed the Facebook posts to be interesting and interactive as studies show that interactive and fun online interventions can enhance well-being [24]. In addition, different modes of delivery, such as audio and video, affect users’ engagement, usability, and enjoyment and can affect mechanisms of change [25].

The content had been prepared before the intervention began and had also been tested in a pilot study. The process of developing and piloting the program has been fully described elsewhere (paper in press). After we piloted the content, we made the necessary revisions. Each post was later re-edited before it was uploaded to the Facebook newsfeed considering real-time occurrences in the group and the ever-changing COVID-19 reality. An overview of the intervention program is presented in Multimedia Appendix 1. At the beginning of the study, participants were asked to set the group’s notification settings to *all posts*, ensuring that they would receive notifications anytime members posted in the group. We posted almost every day, and the participants could read the posts at their convenience.

#### Sample Size Calculation

We used the DWELL index as our primary outcome measure for the calculation. As this is a novel intervention, and with the absence of other relevant data from the literature, we based our calculations on initial data from the pilot study and from the index validation study. We analyzed matched observations from pre- and postintervention questionnaires and used WinPepi to perform the calculations. The mean prepilot DWELL index was 47.5 (SD 18.07), and the mean postpilot DWELL index was 63.57 (SD 11.98). The Pearson correlation coefficient was *r*=0.57. The mean prevalidation DWELL index was 49.2 (SD 20.42), and the mean postvalidation DWELL index was 51.2 (SD 19.11). The Pearson correlation coefficient was *r*=0.81.

For a difference of this magnitude to be detected, we used three different scenarios: (1) a 15-point increase in DWELL score found in the pilot; (2) a 10-point difference for more conservative results; and (3) a 5-point difference assuming that the control group could be contaminated by trial participation, with a smaller effect compared with the intervention group. We assumed that the participants in the RCT might be less engaged with the group than the participants in the pilot study, leading to a reduced expected effect of the program.

We calculated the required sample for power at 80% and 90% and the 2-sided significance level at 5% and 1%. Using 80% power, a 5% 2-sided significance level, and 5 as the difference to be detected, we found that 146 participants (73 pairs) were required as our minimum number to recruit. As online interventions suffer from a high attrition rate [26], we took dropout into account. We decided to recruit twice as many participants as required from our sample size calculation [24,27]. As a result, we needed to include at least 292 participants in the RCT—146 participants in each study group. Recruitment had to be quick as we wanted all participants to join the Facebook group at the same time so that early registrants would not drop out. For that reason, we limited our recruitment time to no longer than 3 weeks (with preference to limit it up to 2 weeks). In this 2- to 3-week recruitment period, we would try to recruit as many participants as possible given that most members of online communities are passive lurkers [28], with a minimum of 146 participants in each group (292 altogether).

#### Study Design

We conducted an RCT in Israel that lasted for 6 weeks, from December 23, 2020, to February 3, 2021, during the third wave of COVID-19. The intervention started just before the Israeli government instructed citizens to stay home for partial lockdown (on December 27, 2020), which later turned into full lockdown (on January 8, 2021). Educational institutions were closed throughout the full lockdown period.

Participant inclusion criteria were Israeli mothers who were aged ≥18 years, had children aged ≤10 years, were literate in Hebrew, used Facebook, and were willing to participate in a Facebook group and answer questionnaires. We targeted mothers as, in Israel, they are often responsible for household management and raising the family [29]. Therefore, by addressing mothers, the intervention could be beneficial to the entire family.

Recruitment was mostly carried out through Facebook and WhatsApp groups (Meta Platforms) and via word of mouth in a voluntary response sample. We advertised our study in a variety of groups with the potential to reach hundreds of thousands of women to maximize the probability of recruiting a diverse population of participants. We added a Google Forms link to the advertisement inviting the mothers who wanted to participate to answer our study’s questionnaire. We told them that they would be asked to answer a second questionnaire again in approximately 6 weeks and that we would add them to the Facebook group sometime in the next 2 months. After recruitment was over, we closed the Google Forms questionnaire and randomized all the participants at once to the intervention and control groups. We used a Microsoft Excel (Microsoft Corp) formula ([=CHOOSE(ROUNDUP(RANK(A2,$A$2:$A$643) /321,0),“intervention,”“control”)]) to randomly assign the participants to the 2 study groups. The participants in the intervention group received a link to the Facebook group. They joined the group in the next day or 2, and afterward, no one could join the group until the intervention was over. The control group participants did not receive anything from us during the time of the intervention. They were not monitored, and we did not have any contact with them until we reached out to them to fill out their second questionnaire at the end of the study. As the Facebook group was closed only to the intervention group, the control group participants could not enter the Facebook group or see any content until the intervention was over. At the end of the intervention, we asked all the participants to fill out the second Google Forms questionnaire. Only after we closed the questionnaire did we open the Facebook group to all Israeli mothers who wanted to join and specifically invited the waitlist control group to join.

As this was an online trial, all the assessments in this study were self-reported. The PhD student carrying out the study managed the Facebook group and performed the outcome assessments, sending the link to the questionnaires to be filled out by the participants and performing the statistical analyses. Thus, blinding was not possible in this study. However, the control group participants were blinded to their allocation. They did not know that we opened the Facebook group or that they were randomized to be the control group.

This RCT was conducted after a pilot study to test our content and experience in managing a Facebook group. The pilot study had a before-and-after design. It was conducted in Israel and lasted for 7.5 weeks, from March 11, 2020, to May 2, 2020, during the first COVID-19 full or partial lockdown. A total of 36 mothers participated in the pilot. They answered online Google Forms questionnaires at the beginning and end of the pilot, and afterward, 11 semistructured telephone interviews were conducted with some of the participants. DWELL was found to be a promising intervention for improving perceptions regarding designing home environments for wellness. These results justified the continuation of the program toward the next phase, the RCT. On the basis of the implications of the pilot study, we made some changes to the methods, which deviated from the clinical trial registration (Textbox 1).

**Changes in methods from the pilot to the next phase of the study.**

- On the basis of the experience from the pilot, and with regard to other online lifestyle interventions [26,30], we decided to shorten the duration of the intervention in the randomized controlled trial (RCT) to 6 weeks instead of the 3 months we had initially planned. The reasons for this decision were as follows:
  The pilot study was successful and beneficial. Its results indicated that this was enough time to detect changes in the Design for Wellness score.
  We hoped to witness a minimal dropout rate in a 6-week intervention. In the pilot study, only 1 participant was lost to follow-up.
  Once we started with recruitment, we needed to do it quickly as we wanted all participants to enter the Facebook group at the same time and we did not want to lose the participants we recruited early on. It would be easier to recruit participants for a 6-week study than for a 3-month study. Furthermore, in a time of COVID-19, we assumed that the participants would have more reservations about committing to a long-term study as there were uncertainties regarding future plans.
  A long-term intervention is harder to replicate. Thus, a 6-week intervention instead of a 3-motnth intervention increased our chances of becoming a role model for other wellness interventions.
- Changing the duration of the intervention to 6 weeks meant that we collected only pre- and postintervention data. We did not ask the intervention group participants to answer midintervention questionnaires, as was initially planned, as it would be unnecessary, would burden the participants, and might increase recall bias.
- Participant inclusion criteria: in the pilot study, the cutoff for participation was mothers of children aged ≤18 years. After the pilot was over, we received feedback from mothers of teenagers indicating that the intervention was more suitable for mothers of young children as parents of adolescents deal with different issues. Therefore, in the RCT, we recruited only mothers of children aged ≤10 years.

### Assessment Tools

#### Objectives 1 and 2: Assessment of DWELL

This study dealt with the new concept of DWELL. To measure our outcomes, we needed a tool that could help us evaluate DWELL. As we could not find any existing tool in the literature, we needed to build and validate a new instrument that could enable us to evaluate our program. Therefore, we developed a short, 5-item online questionnaire to measure the perceptions of participants regarding the impact of home design on wellness. The new DWELL index, with a score ranging from 0 to 100, was calculated through the sum of questions 1 to 5 on the DWELL questionnaire. It was designed to detect changes in the following:

1. Knowledge and awareness in the context of designing a home for a healthy lifestyle, which affects one’s wellness.
2. Intellectual engagement: this level of engagement is about “thinking DWELL.” The intervention will cause participants to be interested in and think about DWELL.
3. Verbal engagement: this level of engagement is about “talking DWELL.” The intervention will cause participants to talk about designing their home environment for wellness.
4. Behavioral engagement: this level of engagement is about “doing DWELL.” The intervention will cause participants to design their home environment for wellness.
5. Self-efficacy regarding DWELL.

We validated the questionnaire on a sample of 613 mothers who answered the questionnaire at the first administration. Of this sample of 613 mothers, 397 (64.8%) answered the questionnaire again at the second administration. Analyses were conducted, and the DWELL questionnaire was found to be a valid tool in a population of mothers and can be used as a valid measure in prevention and wellness interventions. The process of developing and validating the questionnaire has been fully described elsewhere [31].

#### Objective 3: Assessment of Wellness

To measure objective 3, participant wellness, we used two existing questionnaires:

1. The WHO-5: this questionnaire is widely used to assess subjective well-being. It is short and has high internal consistency. It measures general well-being and emphasizes positive feelings [16,32]. The WHO-5 questionnaire has been translated into >30 languages and used in research worldwide [32]. It has also been translated into and validated in Hebrew.
2. The relatively new I COPPE scale integrates important aspects of well-being into a single tool (interpersonal, community, occupational, physical, psychological, economic, and overall well-being). The correlations among these factors have been found to be meaningful and statistically significant [33]. We translated it into Hebrew and, together with the DWELL questionnaire, validated the I COPPE factors that were relevant to this intervention: interpersonal, physical, psychological, and overall well-being.

#### Objectives 4 and 5: Assessment of Engagement

Similar to the overall DWELL index, we developed an overall engagement index with a score ranging from 0 to 100 representing the levels of participation in the Facebook group. This scale was used for the intervention group at the second administration only.

We also used Facebook Insights, which is a free tool provided by Facebook that gives group administrators engagement analyses within their groups [34].

To evaluate participant satisfaction, participants in the intervention group were asked to answer some open-ended questions at the end of the second questionnaire. We asked them to share their experiences in the group, whether they had benefited from it, whether they had learned something new, whether there were topics we missed and they wanted to talk about or issues we discussed and they wanted to delve deeper into, whether there was something in the group that offended them, whether the group was beneficial to them during the COVID-19 lockdown, and whether they had suggestions for improvement and any more comments to share with us.

### Statistical Analyses

All analyses were done using 2-tailed *P* values. Descriptive statistics were computed for explanatory variables and for primary and secondary outcome variables. Sociodemographic variables were measured using questions from validated Hebrew questionnaires [35].

Variables on the DWELL questionnaire were rated on a scale from 0 to 4 and were considered ordinal. All analyses on individual items of the DWELL questionnaire used a nonparametric approach, as did the engagement variables representing levels of participation in the Facebook group, which were on the same scale from 0 to 4. The overall score of the DWELL index, as well as the overall score of the engagement index, was from 0% to 100%, enabling us to use a parametric approach for these analyses. We also used the parametric approach for the wellness variables (I COPPE and WHO-5). I COPPE variables were on a 10-point scale, and the distributions were close to normal, so we considered these variables as continuous and not ordinal. For the WHO-5 variables, we used both parametric and nonparametric tests. Variables were measured on a 6-point scale, and most distributions were close to normal at both time points. We analyzed the WHO-5 variables as continuous and ordinal scales and obtained similar results.

The parametric analyses included paired-sample *t* tests to compare between time 1 and time 2 for each of the study groups and for comparison between the overall DWELL index and the overall engagement index at the end of the study. Independent *t* tests were used to compare between the study groups at each of the time points. They were also used to compare the first-administration overall DWELL index between the participants who answered only the first questionnaire and those who answered both questionnaires in each of the study groups. Finally, they were used for the continuous characteristics to compare between the study groups and between dropouts and completers. Pearson tests were used to assess correlations between the 2 time points for each of the wellness variables and for correlations between the overall engagement index and the overall DWELL index.

The nonparametric analyses included paired Wilcoxon rank sum tests to compare between time 1 (before the intervention) and time 2 (after the intervention) in each of the study groups and Wilcoxon-Mann-Whitney tests (unpaired) to compare between the study groups at each of the time points. Chi-square tests were used for comparison of categorical characteristics between the study groups and between dropouts and completers. Spearman tests were used to assess correlations between levels of individual items of engagement and levels of individual items of the DWELL index at the second administration, as well as to assess correlations between individual items of the DWELL index and between WHO-5 and I COPPE variables at baseline in each of the study groups.

To assess the effectiveness of the intervention, a one-way analysis of covariance was conducted to compare the effect of study group on the overall DWELL score at the end of the study while controlling for the effect of overall DWELL score at the beginning of the study. Multivariable regression analyses using the enter and forward methods were used to statistically predict the dependent variable—the overall DWELL index at the second questionnaire administration using different scenarios from explanatory variables. As the DWELL index was a new variable that we built in the context of this research, we entered all potential confounders into the regression to control for all possible confounding effects.

### Ethical Considerations

This study was approved by the Tel Aviv University Ethics Committee (approval 0000154-4). This trial is registered in the National Institutes of Health Clinical Trials Registry (clinical trial NCT03736525). All participants signed the written informed consent form to take part in the study. They were informed about the purpose of the research, the expected duration of their participation, and that their participation was voluntary and they could discontinue it at any time without it causing them any harm. They were informed that the benefits of the study would be for their entire family. This should compensate them for their time. The questionnaires were not anonymous, but the participants were told that the data would be saved only on the researchers’ computers and that their ID would be removed from any publications. The informed consent allowed for the secondary analysis without having to obtain additional consent.

## Results

### Recruitment, Participation, Randomization, and Completion

There were 686 responders to our Google Forms baseline questionnaire, of whom 643 (93.7%) participated in the study. The other 6.3% (43/686) of responders did not meet the inclusion criteria and were excluded from the study before they could continue to the next section of the questionnaire (the consent form). Of these 43 responders, 3 (7%) were excluded because they were male, 2 (5%) were aged <18 years, 14 (33%) did not have children aged ≤10 years, and 24 (56%) did not have a Facebook profile or did not want to enter a new Facebook group to participate in the study.

Recruitment lasted for an intensive 10 days. Afterward, we closed the Google Forms questionnaire and randomized the 643 participants at once to the intervention or control group. A total of 50.1% (322/643) of the participants were in the intervention group, and 49.9% (321/643) were in the control group. Of the 322 participants in the intervention group, 300 (93.2%) entered the Facebook group, and of these 300 participants, 286 (95.3%) stayed in the group until the end of the study. We also added 16 mothers from personal acquaintance to the Facebook group (including the group administrator—the PhD student carrying out this study). They were not part of the study and did not fill out the study questionnaires. The importance of adding these participants was supported by the conclusions we drew from the pilot study, where most participants did not understand that they were encouraged to upload new posts themselves. In the RCT, we added these participants to the Facebook group to set an example for the other users, demonstrating that it was part of the group’s norm to participate and be active users.

**Figure 1:**
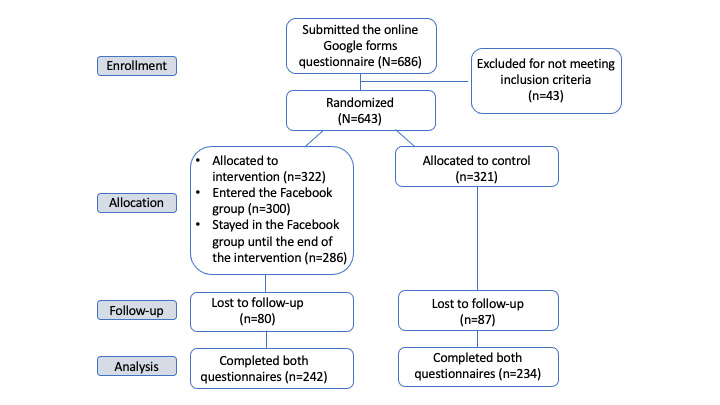
Flowchart of recruitment, randomization, and completion of the study.

Of the 322 participants in the intervention group, 242 (75.2%) answered the second questionnaire, and of the 321 participants in the control group, 234 (72.9%) answered the second questionnaire. The others were lost to follow-up. The flowchart of recruitment, randomization, and completion of the study is presented in Figure 1.

### Participant Characteristics and Comparison Between Study Groups

The mean age of the participants was 36.57 (SD 4.30) years, and the mean number of children aged ≤10 years was 2.06 (SD 0.85). Most of the participants were married (565/636, 87.9%), Jewish (619/635, 97.5%), and secular (495/636, 77.8%). Most of them had attended university or community college (612/638, 95.9%) and had an above-average socioeconomic status (SES; 294/585, 50.3%), with the average gross monthly income per household defined in the questionnaire as New Israeli Shekel 21,000 (approximately US $6000 at the time of questionnaire administration) according to the Israel Central Bureau of Statistics [36]. Participants lived mostly in the central and Hasharon areas of Israel (360/635, 56.7%), and Hebrew was the main mother tongue (584/634, 92.1%). Most of the participants reported good or very good health status (591/634, 93.2%), and most of them were never smokers (424/634, 66.9%) and reported that they had never had someone smoking in their house (473/634, 74.6%). There was no significant difference between the intervention and control groups in any of these characteristics except for education, which was significantly higher in the intervention group (314/319, 98.4% had attended university or community college) than in the control group (298/319, 93.4%; *P*=.002). We also compared baseline characteristics between dropouts (participants who answered only the first questionnaire) and completers (participants who answered both questionnaires). The data are not presented. There was no significant difference between these groups in any of the characteristics except for age, which was significantly higher for the dropouts (mean 36.70, SD 4.30 years; 162/167, 97%) than for the completers (mean 35.29, SD 4.25 years; 465/476, 97.7%; *P*<.001). A description of the sample at baseline and a comparison of characteristics between study groups are presented in Table 1.

**Table 1:** Description of the sample at baseline and comparison of characteristics between study groups (N=643).

		Overall	Intervention (n=322)	Control (n=321)	*P* value	
Age (years), mean (SD)		35.65 (4.30)^a^	35.56 (4.10)^b^	35.75 (4.50)^c^	.59^d^	
Number of children aged ≤10 years, mean (SD)		2.06 (0.85)^e^	2.08 (0.91)^f^	2.03 (0.79)^g^	.46^d^	
**Family status, n (%)**						.80^h^
	Married	565 (87.9)	284 (88.2)	281 (87.5)		
	Not married	78 (12.1)	38 (11.8)	40 (12.5)		
**Religious sector, n (%)**						.62^h^
	Jewish	619 (97.5)	310 (97.8)	309 (97.2)		
	Other	16 (2.5)	7 (2.2)	9 (2.8)		
**Religiosity, n (%)**						.66^h^
	Secular	495 (77.8)	249 (78.5)	246 (77.1)		
	Other	141 (22.2)	68 (21.5)	73 (22.9)		
**Education, n (%)**						.001^h^
	Academic degree	612 (95.9)	314 (98.4)	298 (93.4)		
	Other	26 (4.1)	5 (1.6)	21 (6.6)		
**SES^i^, n (%)**						.65^h^
	Average or below	291 (49.7)	145 (48.8)	146 (50.7)		
	Above average	294 (50.3)	152 (51.2)	142 (49.3)		
**Residence, n (%)**						.73^h^
	Central and Hasharon	360 (56.7)	177 (56)	183 (57.4)		
	Other areas	275 (43.3)	139 (44)	136 (42.6)		
**Mother tongue, n (%)**						.36^h^
	Hebrew	584 (92.1)	288 (91.1)	296 (93.1)		
	Other languages	50 (7.9)	28 (8.9)	22 (6.9)		
**Health status, n (%)**						.44^h^
	Good or very good	591 (93.2)	297 (94)	294 (92.5)		
	Medium or lower	43 (6.8)	19 (6)	24 (7.5)		
**Smoking status, n (%)**						.37^h^
	Smokers or ex-smokers	210 (33.1)	99 (31.4)	111 (34.8)		
	Never smokers	424 (66.9)	216 (68.6)	208 (65.2)		
**Smoking in the house, n (%)**						.34^h^
	Never	473 (74.6)	241 (76.3)	232 (73)		
	Other	161 (25.4)	75 (23.7)	86 (27)		

^a^n=627.

^b^n=312.

^c^n=315.

^d^Independent *t* test.

^e^n=643.

^f^n=322.

^g^n=321.

^h^Chi-square test.

^i^SES: socioeconomic status.

### Objective 1: Primary Outcome of Sum of the DWELL Index

We built an overall DWELL index ranging from 0 to 100 representing levels of DWELL. The overall DWELL score was normally distributed at both the first and second administrations. The mean DWELL score was 50.52 (SD 17.4) at the first administration (N=643) and 57.68 (SD 15.94) at the second administration (476/643, 74%). The primary outcome of the sum of Design for Wellness is presented in Table 2.

In the intervention group, the mean DWELL score was 50.67 (SD 17.75) at the first administration (322/322, 100%) and 61.92 (SD 14.30) at the second administration (242/322, 75.2%). In a paired-sample *t* test, there was a significant improvement of 11.30 (SD 16.34) points (*P*<.001). In the Pearson test, a moderate [37], positive, and significant correlation was found between the 2 time points (*r*=0.52; *P*<.001). We also conducted an independent *t* test to assess the difference between the participants in the intervention group who answered only the first questionnaire (mean 50.81, SD 16.26; 80/322, 24.8%) and those who answered both questionnaires (first administration results: mean 50.62, SD 18.17; 242/322, 75.2%). There was no significant difference between the 2 groups (*P*=.93).

In the control group, the mean DWELL score was 50.37 (SD 17.06) at the first administration (321/321, 100%) and 53.29 (SD 16.37) at the second administration (234/321, 72.9%). In a paired-sample *t* test, there was a significant improvement of 2.31 (SD 14.41) points (*P*=.02). In the Pearson test, a moderate [37], positive, and significant correlation was found between the 2 time points (*r*=0.64; *P*<.001). In the independent *t* test, there was no significant difference between the participants in the control group who answered only the first questionnaire (mean 48.74, SD 15.24; 87/321, 27.1%) and those who answered both questionnaires (first administration results: mean 50.98, SD 17.69; 234/321, 72.9%; *P*=.30).

An independent *t* test was conducted to assess the differences between the 2 study groups at each of the time points. At baseline, there was a nonsignificant mean difference (*P*=.83) of 0.29 points (SD 1.37) between the groups. However, at the end of the study, there was a statistically significant (*P*<.001) mean difference between the study groups of 8.63 points (SD 1.41) in favor of the intervention group.

A one-way analysis of covariance was conducted. The Levene test (*P*=.85) and normality checks were carried out, and the assumptions were met. The intervention group had a significantly higher adjusted mean score of 8.81 points in its overall DWELL score at the end of the study compared with the control group after eliminating the effect of overall DWELL score at the beginning of the study (*F*_1,473_=58.93; *P*<.001).

A multiple regression analysis using the enter method was used to statistically predict the dependent variable—the overall DWELL index at the second questionnaire administration from the respondents’ study group and their overall DWELL index at the first questionnaire administration. These 2 variables were the only ones that showed significance in a univariate analysis (Pearson correlations for the overall DWELL index at the first questionnaire administration and Spearman correlations for the study group). These 2 variables significantly predicted the DWELL index (*F*_2,473_=148.32; *P*<.001; *R*^2^=0.39). A similar analysis was also conducted adding the following potential confounders: age, number of children, health status, religion sector, area of residence, smoking status, smoking-in-the-house status, and SES. Among these variables, age was the only one that showed significance in a univariate analysis (Pearson correlation: *r*=−0.09; *P*=.07). However, it is advised that a variable selection should be more focused on clinical knowledge than statistical selection methods alone. According to “the full model approach,” it is recommended that all candidate variables are included in the model [38]. As the DWELL questionnaire was a new variable that we built in the context of this research, we entered all potential confounders into the regression to control for all possible confounding effects. This model significantly predicted the DWELL index (*F*_10,447_=32.86; *P*<.001; *R*^2^=0.42). However, only the study group (standardized β coefficient=−.27; *P*<.001), the overall DWELL index at the first questionnaire administration (standardized β coefficient=.58; *P*<.001), and age (standardized β coefficient=−.08; *P*=.03) were statistically significant in their contribution to this model. The results are presented in Table 3. A final analysis was conducted with all the previous explanatory variables using the forward method. This model was statistically significant (*F*_2,455_=159.61; *P*<.01; *R*^2^=0.41). In this model, the respondents’ study group (standardized β coefficient=−.27; *P*<.001) and their overall DWELL index at the first questionnaire administration (standardized β coefficient=.58; *P*<.001) were the only variables that statistically improved the prediction.

**Table 2:** Sum of Design for Wellness (DWELL) index and comparison between study groups and between time points.

Time point		Overall	Intervention	Control	Mean (SD)^a^		*P* value		Mean (SD)^b^		*P* value	
**Sum of DWELL index, mean (SD)**						11.30 (16.34)		<.001		2.31 (14.41)		.02
	T1^c^	50.52 (17.4)	50.67 (17.75)	50.37 (17.06)								
	T2^d^	57.68 (15.94)	61.92 (14.30)	53.29 (16.37)								
**Mean difference (SD)^e^**												
	T1	—^f^	0.29 (1.37)	0.29 (1.37)	—		.83		—		—	
	T2	—	8.63 (1.41)	8.63 (1.41)	—		<.001		—		—	

^a^Paired-sample *t* test to compare between time 1 and time 2 for the intervention group.

^b^Paired-sample *t* test to compare between time 1 and time 2 for the control group.

^c^T1: first questionnaire.

^d^T2: second questionnaire.

^e^Independent *t* test to compare between the study groups at each of the time points.

^f^Not available.

**Table 3:** Coefficients for the overall Design for Wellness (DWELL) score at the second questionnaire administration using the enter method.

Variable	Standardized β coefficient	95% CI	*P* value
DWELL score at first administration	.58	0.452 to 0.577	<.001
Study group	−.27	−10.667 to −6.207	<.001
Age	−.08	−0.588 to −0.034	.03
Religion sector	−.04	−4.565 to 1.478	.32
SES^a^	.05	−0.283 to 1.531	.18
Residence	.000	−0.558 to 0.560	.99
Health status	−.05	−2.973 to 0.481	.16
Smoking status	−.01	−1.991 to 1.357	.71
Smoking in the house	.02	−0.510 to 0.894	.59
Number of children aged ≤10 years	.03	−0.837 to 1.938	.44

^a^SES: socioeconomic status.

### Objective 2: Secondary Outcomes Regarding Individual Items of the DWELL Questionnaire

In the intervention group, each of the five individual DWELL variables—(1) knowledge and awareness, (2) intellectual engagement, (3) verbal engagement, (4) behavioral engagement, and (5) self-efficacy—improved significantly in the Wilcoxon tests from the first to the second questionnaire. In the control group, 3 of the 5 items improved significantly (knowledge and awareness, intellectual engagement [thinking DWELL], and behavioral engagement [doing DWELL]). There was no change in the verbal engagement (talking DWELL) question or the self-efficacy question.

In the Mann-Whitney tests comparing the intervention and control groups, there was no significant difference in any of the 5 DWELL variables in the baseline questionnaire. However, all 5 variables were significantly higher in the intervention group than in the control group at the second administration.

Descriptive statistics of the 5 DWELL items for each of the study groups and the results of the Wilcoxon signed rank tests and Mann-Whitney tests are presented in Table 4.

**Table 4:** Descriptive statistics of the 5 Design for Wellness (DWELL) variables and Wilcoxon and Mann-Whitney tests for comparing DWELL variables between the 2 time points and between the 2 study groups.

Study group, DWELL item, and time point				Participants, n (%)		Mean (SD)		Median		Mode		Range		*P* value	
**Intervention group (n=322)**															
	**Knowledge and awareness^a^**														<.001^b^
		T1^c^	321 (99.7)		2.68 (0.95)		3		3		0-4				
		T2^d^	242 (75.2)		3.26 (0.68)		3		3		1-4				
	**Thinking DWELL^e^**														<.001^b^
		T1	322 (100)		1.52 (1.06)		1		1		0-4				
		T2	241 (74.8)		2.10 (0.91)		2		2		0-4				
	**Talking DWELL^f^**														<.001^b^
		T1	322 (100)		1.48 (1.03)		1		2		0-4				
		T2	241 (74.8)		1.92 (0.93)		2		2		0-4				
	**Doing DWELL^g^**														<.001^b^
		T1	322 (100)		1.69 (0.96)		2		2		0-4				
		T2	241 (74.8)		2.22 (0.81)		2		2		0-4				
	**Self-efficacy of DWELL^h^**														.03^b^
		T1	322 (100)		2.78 (0.80)		3		3		0-4				
		T2	242 (75.2)		2.90 (0.74)		3		3		1-4				
**Control group (n=321)**															
	**Knowledge and awareness**														.005^b^
		T1	320 (99.7)		2.77 (0.86)		3		3		0-4				
		T2	234 (72.9)		3.00 (0.83)		3		3		0-4				
	**Thinking DWELL**														.01^b^
		T1	320 (99.7)		1.45 (1.02)		1		1		0-4				
		T2	234 (72.9)		1.61 (0.94)		2		1		0-4				
	**Talking DWELL**														.62^b^
		T1	318 (99.1)		1.41 (1.04)		1		1		0-4				
		T2	233 (72.6)		1.46 (0.97)		1		1		0-4				
	**Doing DWELL**														.02^b^
		T1	320 (99.7)		1.73 (0.97)		2		2		0-4				
		T2	234 (72.9)		1.90 (0.93)		2		2		0-4				
	**Self-efficacy of DWELL**														.28^b^
		T1	321 (99.7)		2.74 (0.78)		3		3		0-4				
		T2	234 (72.9)		2.70 (0.85)		3		3		0-4				

^a^Intervention versus control group: *P*=.25 at time 1 and *P*<.001 at time 2 (Mann-Whitney tests to compare between the study groups at each of the time points).

^b^Wilcoxon tests to compare between time 1 and time 2 for each of the study groups.

^c^T1: first questionnaire.

^d^T2: second questionnaire.

^e^Intervention versus control group: *P*=.46 at T1 and *P*<.001 at T2 (Mann-Whitney tests to compare between the study groups at each of the time points).

^f^Intervention versus control group: *P*=.26 at T1 and *P*<.001 at T2 (Mann-Whitney tests to compare between the study groups at each of the time points).

^g^Intervention versus control group: *P*=.58 at T1 and *P*<.001 at T2 (Mann-Whitney tests to compare between the study groups at each of the time points).

^h^Intervention versus control group: *P*=.43 at T1 and *P*=.005 at T2 (Mann-Whitney tests to compare between the study groups at each of the time points).

### Objective 3: Secondary Outcomes Regarding Wellness (as Measured Using the I COPPE and WHO-5 Instruments)

#### Individual Items of I COPPE

Descriptive statistics of I COPPE variables (regarding interpersonal, physical, psychological, and overall well-being) and results for independent and dependent *t* tests for comparing the differences between the 2 study groups for each variable at each time point and for comparing the differences between the 2 time points for each of the study groups separately are presented in Table 5.

In the intervention group, overall well-being increased from the first to the second administration (from mean 7.41, SD 1.57 to mean 7.61, SD 1.41; 239/322, 74.2%; *P*=.01 [mean results from paired samples]), and physical well-being also increased (from mean 5.84, SD 2.20 to mean 6.06, SD 2.06; 242/322, 75.2%; *P*=.04). There was no significant difference in interpersonal or psychological well-being between the groups. In the control group, there was no difference in any of the I COPPE variables between the first and second administrations. There was only a close to significant increase in the physical well-being item (from mean 6.02, SD 2.25 to mean 6.21, SD 2.14; 234/321, 72.9%; *P*=.06). There was no significant difference between the 2 study groups in any of the variables at the first or second questionnaire administration.

**Table 5:** Descriptive statistics and *t* tests for I COPPE variables in each of the study groups at both questionnaire administrations.

Study group, I COPPE item, and time point			Participants, n (%)	Mean (SD)^a^	Median	Mode	Range	*P* value	
**Intervention group (n=322)**									
	**Overall well-being^b^**								.01^c^
		T1^d^	322 (100)	7.46 (1.51)	8	8	2-10		
		T2^e^	239 (74.2)	7.61 (1.41)	8	8	1-10		
	**Interpersonal well-being^f^**								.48^c^
		T1	322 (100)	7.79 (1.71)	8	9	1-10		
		T2	242 (75.2)	7.83 (1.75)	8	9	0-10		
	**Physical well-being^g^**								.04^c^
		T1	322 (100)	5.93 (2.18)	6	7	0-10		
		T2	242 (75.2)	6.06 (2.06)	6	7	0-10		
	**Psychological well-being^h^**								.23^c^
		T1	322 (100)	7.01 (1.93)	7	8	0-10		
		T2	242 (75.2)	6.79 (1.99)	7	8	0-10		
**Control group (n=321)**									
	**Overall well-being**								.56^c^
		T1	321 (100)	7.48 (1.50)	8	8	1-10		
		T2	233 (72.6)	7.63 (2.17)	8	8	2-10		
	**Interpersonal well-being**								.92^c^
		T1	321 (100)	7.55 (1.83)	8	9	1-10		
		T2	234 (72.9)	7.67 (1.86)	8	9	1-10		
	**Physical well-being**								.06^c^
		T1	321 (100)	6.00 (2.17)	6	7	0-10		
		T2	234 (72.9)	6.21 (2.14)	7	7	0-10		
	**Psychological well-being**								.43^c^
		T1	321 (100)	6.85 (1.89)	7	8	1-10		
		T2	234 (72.9)	6.90 (1.91)	7	8	0-10		

^a^Mean of the total group, which differs slightly from the mean in the paired *t* test, which included only participants with responses for 2 time points.

^b^Intervention versus control group: *P*=.87 at time 1 and *P*=.88 at time 2 (independent *t* test to compare between the study groups at each of the time points).

^c^Paired-sample *t* test to compare between time 1 and time 2 for each of the study groups.

^d^T1: first questionnaire.

^e^T2: second questionnaire.

^f^Intervention versus control group: *P*=.09 at T1 and *P*=.31 at T2 (independent *t* test to compare between the study groups at each of the time points).

^g^Intervention versus control group: *P*=.69 at T1 and *P*=.43 at T2 (independent *t* test to compare between the study groups at each of the time points).

^h^Intervention versus control group: *P*=.29 at T1 and *P*=.55 at T2 (independent *t* test to compare between the study groups at each of the time points).

#### Individual Items of the WHO-5

We analyzed the WHO-5 variables as continuous and ordinal scales and obtained similar results. The data are presented in Table 6 for the continuous analyses only.

Descriptive statistics of the WHO-5 variables and results for independent and dependent *t* tests for comparing the differences between the 2 study groups for each variable at each time point and for comparing the differences between the 2 time points for each of the study groups separately are also presented in Table 6. Each of the questions referred to the last 2 weeks before the time of administration.

In the intervention group, participants reported feeling less calm and relaxed at follow-up (from mean 2.65, SD 1.06 to mean 2.46, SD 1.03; 240/322, 74.5%; *P*=.006 mean results from paired samples) and also that their daily lives had been less filled with things that interested them (from mean 2.58, SD 1.17 to mean 2.40, SD 1.15; 242/322, 75.2%; *P*=.02). There was a close to significant decrease in their report of being cheerful and in good spirits (from mean 2.94, SD 0.99 to mean 2.82, SD 0.95; 241/322, 74.8%; *P*=.07) and a close to significant increase in their report of being fresh and rested on waking (from mean 1.48, SD 1.13 to mean 1.61, SD 1.14; 242/322, 75.2%; *P*=.06). This result was significant on the ordinal scale using the Wilcoxon signed rank test (*P*=.03). There was no difference in how active and vigorous they had felt (242/322, 75.2%; *P*=.84).

In the control group, the participants reported feeling less cheerful and in good spirits at follow-up (from mean 3.05, SD 0.93 to mean 2.83, SD 1.03; 234/321, 72.9%; *P*=.001), less calm and relaxed (from mean 2.72, SD 1.10 to mean 2.47, SD 1.01; 231/321, 72%; *P*=.001), and less active and vigorous (from mean 2.44, SD 1.01 to mean 2.15, SD 0.96; 234/321, 72.9%; *P*<.001) and that their daily lives had been less filled with things that interested them (from mean 2.60, SD 1.10 to mean 2.36, SD 1.22; 234/321, 72.9%; *P*=.001). There was a close to significant increase in their report of being fresh and rested on waking (from mean 1.61, SD 1.16 to mean 1.73, SD 1.18; 233/321, 72.6%; *P*=.08).

There was no significant difference between the 2 study groups for any of the variables in the first or second questionnaires.

We also assessed correlations between individual items of the DWELL questionnaire and between WHO-5 and I COPPE variables at baseline for each of the study groups. In both study groups, most of the correlations were not significant in Spearman tests, and those that were significant were low [37] (Spearman correlation coefficient ranging between 0.11 and 0.24).

**Table 6:** Descriptive statistics and *t* tests for the 5-item World Health Organization Well-Being Index (WHO-5) variables in each of the study groups at both questionnaire administrations.

Study group, WHO-5 item, and time point				Participants, n (%)	Mean (SD)^a^	Median		Mode		Range		*P* value	
**Intervention group (n=322)**													
	**Good spirits^b^**												.07^c^
		T1^d^	321 (99.7)		2.94 (0.99)	3	3		0-5				
		T2^e^	242 (75.2)		2.82 (0.95)	3	3		0-4				
	**Calm and relaxed^f^**												.006^c^
		T1	321 (99.7)		2.68 (1.07)	3	3		0-5				
		T2	241 (74.8)		2.46 (1.03)	3	3		0-5				
	**Active and vigorous^g^**												.84^c^
		T1	322 (100)		2.27 (0.99)	2	2		0-5				
		T2	242 (75.2)		2.27 (0.97)	2	2		0-4				
	**Fresh and rested^h^**												.06^c^
		T1	322 (100)		1.54 (1.13)	1	1		0-4				
		T2	242 (75.2)		1.61 (1.14)	2	1		0-4				
	**Interest in life^i^**												.02^c^
		T1	322 (100)		2.61 (1.14)	3	3		0-5				
		T2	242 (75.2)		2.40 (1.15)	2	3		0-5				
**Control group (n=321)**													
	**Good spirits**												.001^c^
		T1	321 (100)		2.98 (0.98)	3	3		1-5				
		T2	234 (72.9)		2.83 (1.03)	3	3		0-5				
	**Calm and relaxed**												.001^c^
		T1	319 (99.4)		2.68 (1.09)	3	3		0-5				
		T2	232 (72.3)		2.47 (1.01)	2	2		0-4				
	**Active and vigorous**												<.001^c^
		T1	321 (100)		2.34 (1.03)	2	2		0-5				
		T2	234 (72.9)		2.15 (0.96)	2	2		0-4				
	**Fresh and rested**												.08^c^
		T1	321 (100)		1.61 (1.15)	2	2		0-5				
		T2	233 (72.6)		1.73 (1.18)	2	2		0-5				
	**Interest in life**												.001^c^
		T1	321 (100)		2.58 (1.13)	3	3		0-5				
		T2	234 (72.9)		2.36 (1.22)	2	2		0-5				

^a^Mean of the total group, which differs slightly from the mean in the paired *t* test, which included only participants with responses for 2 time points.

^b^Intervention versus control group: *P*=.66 in time 1 and *P*=.91 in time 2 (independent *t* test to compare between the study groups at each of the time points).

^c^Paired-sample *t* test to compare between time 1 and time 2 for each of the study groups.

^d^T1: first questionnaire.

^e^T2: second questionnaire.

^f^Intervention versus control group: *P*=.98 in T1 and *P*=.99 in T2 (independent *t* test to compare between the study groups at each of the time points).

^g^Intervention versus control group: *P*=.38 in T1 and *P*=.18 in T2 (independent *t* test to compare between the study groups at each of the time points).

^h^Intervention versus control group: *P*=.42 in T1 and *P*=.27 in T2 (independent *t* test to compare between the study groups at each of the time points).

^i^Intervention versus control group: *P*=.74 in T1 and *P*=.73 in T2 (independent *t* test to compare between the study groups at each of the time points).

### Objectives 4 and 5: Secondary Outcomes Regarding Engagement With the Facebook Group and DWELL

The overall engagement score was normally distributed (mean 35.64, SD 19.22; 242/322, 75.2%). When comparing the overall DWELL index (mean 61.92, SD 14.30) with the overall engagement index at the second administration (242/322, 75.2%), there was a mean significant difference of 26.28 (SD 19.24) points (*P*<.001) in a paired-sample *t* test.

The engagement score had no correlation with the DWELL score at the first administration (*r*=0.008 in a Pearson test; *P*=.90) and a low [37], positive, and significant correlation with the DWELL score at the second administration (*r*=0.37; *P*<.001).

Spearman correlations between levels of engagement and items of the DWELL index at the second administration were mostly low [37], positive, and significant (Spearman correlation coefficient ranging between 0.13 and 0.34). Engagement using reactions such as *like* had a low and close to significant correlation with self-efficacy regarding DWELL (*r*_s_=0.12; *P*=.06). Higher levels of engagement, such as commenting on posts, had low and not significant correlations with intellectual engagement of “thinking DWELL” (*r*_s_=0.96; *P*=.14) and with self-efficacy regarding DWELL (*r*_s_=0.109; *P*=.09). The highest level of engagement, creating new posts, had low and not significant correlations with behavioral engagement of “doing DWELL” (*r*_s_=0.77; *P*=.24) and with self-efficacy regarding DWELL (*r*_s_=0.099; *P*=.12). However, only a few participants reported high levels of participation as the engagement score between the first and second quartiles (25%-75%) ranged between 20 and 45 (242/322, 75.2%).

**Figure 2:**
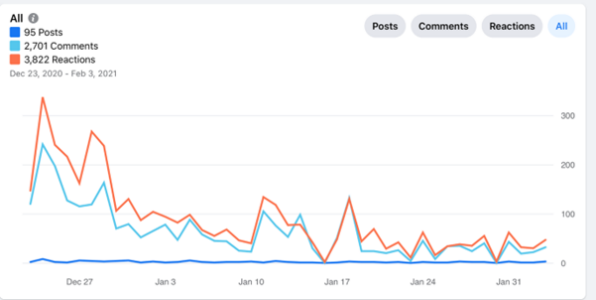
Facebook Insights data on engagement with the Facebook group intervention.

Facebook Insights also provided information regarding engagement with the group during the 6 weeks of intervention. Altogether, there were 95 posts, 2701 comments, and 3822 reactions. These data are presented in Figure 2. The very high engagement in the first few days was because many participants introduced themselves at once. After we opened the Facebook group, we asked the participants to introduce themselves and the reasons why they joined the Facebook group. Of the 322 participants, approximately 90 (28%) commented on this post sharing who they were and what they were expecting to gain from this group. There were >200 comments on this post as the group administrator (the PhD student carrying out this study) answered each one and the participants also commented on each other. The group remained very active for the entire intervention as posts were uploaded daily and all of them received comments and reactions.

### Open-Ended Feedback About the Facebook Group From Intervention Group Participants

Of the 242 participants in the intervention group who answered both questionnaires, 210 (86.8%) answered at least one of the open-ended questions.

The feedback we received from the participants was mostly good. Most of them (141/242, 58.3) loved the group and enjoyed participating. They felt that the group was very supportive and accepting, with lots of good ideas, positive interactions, and freedom from judgment.

Many participants (128/242, 52.9%) shared that they had learned something new in the group. However, even if they thought that the ideas in the group were not new to them, they still shared that it made them think about DWELL issues and raised their awareness regarding DWELL. They shared that they thought about their own homes and embraced some of the recommendations. Some of the participants (4/242, 1.7%) mentioned that they had discussions about the ideas in the group with their husbands.

Regarding participation in the group during the COVID-19 lockdown, almost half of the participants (119/242, 49.2%) thought that DWELL was very beneficial as it helped them and their families live healthier at a time when they had to stay home. However, some (29/242, 12%) said that, during the lockdown, they had less time than usual and could not find the time to be on Facebook. Some also added that, in their opinion, DWELL content is always relevant and that they did not think it was more beneficial during the COVID-19 lockdown than during any other time.

## Discussion

### Principal Findings and Comparison With Previous Work

This study dealt with the new concept of DWELL. DWELL was found to be a beneficial intervention. This was supported by a robust quantitative questionnaire and by qualitative, open-ended questions. A Facebook group was found to be an effective platform to deliver this online intervention.

Our primary outcome, the overall DWELL index, showed no significant difference between the study groups at baseline (*P*=.83), but at the end of the study, there was a statistically significant (*P*<.001) mean difference of 8.63 (SD 1.41) points in favor of the intervention group. This difference was preserved while controlling for the effect of the overall DWELL index at baseline. The overall DWELL index increased significantly by 11.30 points in the intervention group from the beginning to the end of the study. In the control group, there was also a significant but much smaller increase of 2.31 points. The exact same 2.31-point increase was also observed in the validation study of the DWELL questionnaire, where the participants answered the DWELL questionnaire twice without receiving an intervention [31]. This 2-point increase in the DWELL score can be explained by the “mere measurement” effect, which indicates that, by simply asking participants questions, it is possible to influence their behaviors [39,40].

The specific items of the DWELL questionnaire also indicated positive results as all 5 measures (knowledge and awareness, 3 levels of engagement, and self-efficacy regarding DWELL) increased significantly in the intervention group from the beginning to the end of the study. Although there was no significant difference in any of the specific questions of the DWELL index at baseline between the study groups, all 5 variables were significantly higher in the intervention group at the second administration than in the control group.

Qualitative feedback from participants in the intervention group strengthened the positive results as most of them found the group to be beneficial.

The study’s positive results are consistent with those of other studies that strongly indicate that nudge strategies hold promise in public health interventions and have the potential to influence at the population level [41,42]. Although evidence to date on nudge research is growing, most of these studies suffer from inadequate design. This study contributes to the nudging literature as there is a need for more RCTs and high-quality studies are called for [6]. Furthermore, there is a need for more real-world research because of the limitation of few nudging interventions outside of the laboratory setting [41,42].

Most of the nudging interventions that we found in the public health literature dealt with one component of healthy behaviors, mainly dieting and food consumption behaviors but also physical activity, hygiene, and littering [6,41-46]. The presence of multiple risk behaviors has been shown to have an additive or synergistic negative effect on health as most individuals engage in multiple unhealthy lifestyle behaviors [47]. A healthy lifestyle, according to the World Health Organization, is creating a better environment that contributes to the well-being and enjoyment of life while addressing different aspects of behavior such as physical activity, healthy eating, and exposure to tobacco [48]. Health promotion interventions that simultaneously target the improvement of multiple risk behaviors could have a greater impact on individuals’ health than interventions that target single risk behaviors [47]. In this work, the intervention addressed the design of the home environment for multiple components of health behavior, such as healthy eating, physical activity, tobacco-free environment, hygiene, family conversations regarding wellness issues, and reduction of stress. The full overview of the intervention program is presented in Multimedia Appendix 1.

Although the primary and secondary outcomes of DWELL (objectives 1 and 2) were found to be beneficial, we did not find an improvement in the secondary outcome regarding participants’ well-being (objective 3). For lifestyle interventions, as per the Health Belief Model [49], behavior change occurs before the target outcome that concerns health status [50]. In the I COPPE [33] and WHO-5 [16,32] questionnaires, there were no significant differences between the 2 study groups for any of the variables at the first or second questionnaire administrations. Regarding the specific items of the WHO-5 questionnaire, the participants in both the intervention and control groups reported being less calm and relaxed and that their daily lives had been less filled with things that interested them (*P*<.05). However, participants in the control group only also reported being less cheerful and in good spirits and less active and vigorous (*P*<.01). The results of the well-being questions may have been biased by the COVID-19 lockdown. The WHO-5 questionnaire [16,32] refers to the well-being of responders over the last 2 weeks before the completion of the questionnaire. In this study, the first questionnaire was administered before the third lockdown began. The second questionnaire was administered during the third full lockdown. It is reasonable to assume that the participants’ spirits were affected by the lockdown, causing them to be less calm and relaxed as they had probably needed to take care of their children and manage their homes while continuing to work. In addition, they were less interested in things in their daily lives as they had probably worked less than usual and had less time for themselves to do things that interested them. However, the participants in the intervention group felt fresher and more rested on waking (this result was statistically significant in the nonparametric test, *P*=.03, and close to significant in the parametric test, *P*=.06). It is possible that, while staying home, they did not need to manage their daily activities, such as driving to work, taking their children to and from school or the nursery, and looking for afternoon and weekend activities, causing them to feel more rested. The overall and physical well-being of the intervention group also increased significantly (*P*<.05), whereas no change was detected in the control group for any of the I COPPE variables. It is possible that DWELL helped the intervention group participants during the COVID-19 lockdown. The program aimed to help participants design their home environment for wellness, and this was a unique time when everyone stayed home more. The DWELL intervention helped the participants eat better, stay active at home, reduce the level of stress, and preserve hygiene at a time when the participants needed it the most and looked for ways to be healthier at home. Mothers, who were at home more than usual, had the opportunity to implement ideas. Furthermore, the intervention was online, enabling the participants to see/engage with the content without exposing themselves to unnecessary risks.

Facebook Insights indicated that the group was very active relative to a new group and the short 6-week duration. The high engagement started from the first welcoming post as many of the participants introduced themselves to the group in a revealing and open-hearted way. It looked as if the participants had been waiting for such a group to emerge, enabling them to open up and share their thoughts, difficulties, and dilemmas regarding designing a healthy home environment. The participants shared that they found this group to be supportive and not judgmental and appreciated the chance to participate in a platform that enabled them to share and also learn from others.

Although the group was active, we only found a low, positive, and significant correlation between the overall DWELL score and the overall engagement score at the second questionnaire administration (objective 4; *r*=0.37; *P*<.001). This indicates that being more engaged with the group was correlated with having a higher DWELL score, but this relationship was weak. The same low, positive, and significant correlations were also found between levels of engagement and specific items of the DWELL questionnaire (Spearman correlation coefficient ranging between 0.13 and 0.34; objective 5). We did not find a significant correlation between the overall DWELL score at the first questionnaire administration and the overall engagement score at the end of the study (*r*=0.008; *P*=.90). This indicates that the participants who were more engaged in the group did not have an initially higher DWELL score when they joined the study. The mean significant difference of 26.281 (SD 19.24) points between the overall DWELL score and the overall engagement score indicated that participants who were not active in the group still followed the posts and benefited from the group. Users in online communities can participate in an active and public manner, posting and commenting, or in a passive “lurking” and not public manner, regularly consuming content without posting themselves [28,51]. Most of the members of online communities are passive lurkers, but they can benefit cognitively and socially through a vicarious learning process of observing others’ learning [28]. The results of this study strengthen this claim.

This study had a relatively large sample size of 643 participants and a high response rate, both in the intervention group (242/322, 75.2%) and in the control group (234/321, 72.9%). Online interventions usually suffer from high attrition rates as many studies report 10% to 25% retention rates [26,52,53]. Online studies that report higher retention rates, such as 50% to 80%, usually involve incentives to participants [54,55]. In our study, the participants in the intervention group had access to the intervention, but the waitlist control group did not receive anything for their participation until they filled out both the pre- and postintervention questionnaires. Only then they could join the Facebook group. None of the participants received any other rewards for taking part other than access to the intervention. Hence, we consider this to be a relatively high response rate. We calculated the overall DWELL index between the participants who answered only the first questionnaire and those who answered both questionnaires (first administration results) for each of the study groups and found no significant difference. This analysis indicates a lack of response bias between the groups.

### Limitations

This study has some limitations. First, we advertised the study in a variety of Facebook groups to reach a diverse population of women who fit the criteria. Even so, our participants were found to be a nonrepresentative population of women. Most of them had attended university or community college (612/638, 95.9%) and had an above-average SES (294/585, 50.3%). They mostly lived in the central and Hasharon areas of Israel (360/635, 56.7%) and reported a good or very good health status (591/634, 93.2%). There is evidence that education and health status influence participation in studies [56]. Populations that experience disadvantages typically have lower rates of participation in research [51]. Although the participants were not sufficiently representative of the population of Israeli mothers, the overall DWELL index was normally distributed at both questionnaire administrations, which indicates an adequate variance of the scale.

Second, the intervention was conducted during the COVID-19 partial and then full lockdown. This had some advantages as the intervention was designed to help families create a healthier home environment at a time when they were constantly at home and needed it the most. The intervention was provided on Facebook, an online platform that enabled them to participate during the lockdown without exposing themselves to risks. However, the timing could also bias the results of the study as we cannot know how the intervention would have been accepted on regular days. Most mothers who answered the open-ended questionnaire shared with us that they thought that it would not have made any difference. We created the content of the intervention before the first COVID-19 outbreak and only made some minor adjustments in real time. Hence, the content is always relevant, not just for times of lockdown. COVID-19 probably also biased the results of our secondary outcome, the participants’ wellness. Some of the participants shared in the open-ended questions that being in lockdown affected their spirits. They asked us to acknowledge that their responses to the well-being questions were biased by it. Finally, the participants joined the study before the third lockdown began. Although some mothers were more available, others told us in the open-ended questions that they could not find the time to participate or implement ideas.

Third, we used a self-report questionnaire to measure our end points, with a voluntary response sample. Online questionnaires can suffer from self-selection bias, and in addition, as all assessments were self-reported, reporting errors may have occurred. Even so, self-report questionnaires save time and money, provide access to diverse populations, enable researchers to collect large amounts of data with depth and details in a short period, and capture a range of thoughts and views [57,58]. Although there are many advantages to using questionnaires, it would have been interesting to measure actual changes in the participants’ homes and not rely only on self-report measurements. Future studies should investigate this, taking into consideration that this type of study will be more expensive, will be harder to conduct and recruit for, and will narrow the potential recruitment geographic reach as opposed to an online study with online questionnaires.

Fourth, this study lacked a long-term follow-up. It is possible that a longer follow-up might have produced better wellness outcomes.

Fifth, we do not have information regarding the environment of each of the participants. In future studies, it would be important to devise ways to assess the health-promoting aspects of home environments at baseline to see whether that variable makes a difference in the study results.

Finally, this study was conducted with Hebrew-speaking, educated Israeli mothers. Future studies should examine the effectiveness of this intervention in other cultures and populations and in languages other than Hebrew.

### Conclusions

DWELL is an online Facebook intervention aimed at improving the perceptions of participants regarding designing their home environment for wellness. The dynamic platform produced social learning, enabling the participants to be engaged with different aspects of wellness issues, and the content was delivered in reliable and enjoyable ways. The intervention was effective in increasing the knowledge and awareness, verbal engagement, intellectual engagement, behavioral engagement, and self-efficacy of a population of educated Israeli mothers of young children regarding DWELL. The intervention was also found to be effective during emerging viral threats such as COVID-19. This study contributes to nudging research and research on Facebook interventions for promoting well-being. As the intervention was found to be beneficial, it may become a prototype for similar health promotion/lifestyle interventions elsewhere.

## Appendix

Multimedia Appendix 1 An overview of the intervention program.

Multimedia Appendix 2 CONSORT-EHEALTH checklist (V 1.6.1).

## Abbreviations

DWELL: Design for Wellness
I COPPE: Interpersonal, Community, Occupational, Physical, Psychological, Economic and Overall Well-Being
RCT: randomized controlled trial
SES: socioeconomic status
WHO-5: 5-item World Health Organization Well-Being Index

## Data Availability

The data sets generated and analyzed during this study are available from the corresponding author upon reasonable request.
