# Supplementary material for: Improving Knowledge, Engagement, and Self-Efficacy in the Creation of Healthy Home Environments for Mothers Using a Facebook Intervention (Design for Wellness): Randomized Controlled Trial
Source: J Med Internet Res. 2023 Nov 7;25:e46640. doi: 10.2196/46640 (PMC10664014; doi:10.2196/46640)
Supplement: Multimedia Appendix 1 [file jmir_v25i1e46640_app1.docx]

Appendix 1. An overview of intervention program

| **Post Number** | **Objectives** | **Contents** | **Format** |
| --- | --- | --- | --- |
| 1 | Instructions | Turn on notifications to “all posts” | An explanation and a photo |
| 2 | Create welcoming environment | Introduction, purpose of the group | An explanation and a question |
| 3 | Create a sense of community and belonging | Introduction, self-presentation, purpose of the group | A personal story |
| 4 | Theory to understand DWELL and the need for this group | Research information.  People are driven by instincts | A question and an answer |
| 5 | Theory to understand DWELL and the need for this group | Research information.  Design environment is inevitable and influences our decisions | A poll |
| 6 | Theory to understand DWELL and the need for this group | Research information.  Design environment is inevitable and influences our decisions | A question and an answer |
| 7 | Theory to understand DWELL and the need for this group | Research information.  People stick with default choices | A question and an answer |
| 8 | A summary for theory (posts 4-7).  From theory to practice | Repeating important messages and conclusions | Questions and answers |
| 9 | Share ideas, create engagement | A recommendation for DWELL to encourage healthy eating | A personal story and a picture |
| 10 | Share knowledge and ideas, create engagement | A recommendation for DWELL to encourage hygiene | A picture |
| 11 | Share knowledge, create openness and sharing | Family conversations about wellness | A personal story |
| 12 | Create engagement and set DWELL goals for new year | New years’ resolution for DWELL | A text and a question |
| 13 | Share knowledge | A recommendation for DWELL to encourage physical activity | A YouTube video and a personal video for personal example |
| 14 | Share ideas, create engagement | A recommendation for DWELL to encourage healthy eating | A personal story and a picture |
| 15 | Share knowledge, create engagement, raise awareness of DWELL | Research information regarding smoking at balconies | A picture and a question |
| 16 | Create engagement | Raise awareness regarding proper ways to wash fruits and vegetables | A poll |
| 17 | Share knowledge, create engagement, raise awareness of DWELL | A recommendation for DWELL to properly wash fruits and vegetables | A text |
| 18 | Share knowledge, create engagement | A recommendation for DWELL to increase wellbeing and reduce stress | A text and a YouTube song |
| 19 | raise awareness of DWELL | Playlist of quiet songs the participants chose in the previous post | A text and a YouTube and Spotify playlists |
| 20 | Share knowledge, create engagement, raise awareness of DWELL | A recommendation for DWELL to encourage healthy eating and family conversation | A personal story and pictures |
| 21 | Create engagement | DWELL for stress management | questions |
| 22 | Create engagement | Raise interest toward physical health | A question |
| 23 | Share knowledge | A recommendation for DWELL to encourage physical and mental health | A video and an explanation |
| 24 | Create engagement | Encourage them to bring DWELL solutions for corona masks storage at home | A question |
| 25 | Create engagement, raise self-efficacy | A challenge | Take and upload a picture, comment to others |
| 26 | Create engagement, encourage participants to share content with their young children | Family conversations about wellness and physical activity | A rhyme and a question |
| 27 | Share knowledge, create engagement | A recommendation for DWELL to encourage 2 min brushing teeth | A picture |
| 28 | Create engagement and a sense of community, share ideas | Ideas for inside activities.  Encourage physical activity and enhance interpersonal wellbeing | Questions |
| 29 | Create engagement | DWELL to encourage healthy eating | Questions |
| 30 | Share knowledge, create engagement | Research information.  A recommendation for DWELL to encourage healthy eating habits | A poll |
| 31 | Share knowledge, create engagement | A recommendation for DWELL to encourage healthy eating habits and family conversations on this issue | A personal story and a video |
| 32 | Share knowledge, create engagement | A recommendation for DWELL to encourage proper food storage | A text and a question |
| 33 | Share knowledge, create engagement | DWELL to prevent third hand smoking | A text and questions |
| 34 | Share ideas, create engagement | A recommendation for DWELL to encourage healthy eating | A personal story and pictures |
| 35 | Share knowledge, create engagement, relate to the spirit of the holiday | A recommendation for DWELL to store properly nuts and almonds | A text and questions |
| 36 | Share knowledge | A recommendation for DWELL to encourage physical health | A video and an explanation |
| 37 | Share ideas, create engagement | A recommendation for DWELL to encourage family conversations through children’s books | Text and pictures |
| 38 | Share knowledge, create engagement | Research information.  A recommendation for DWELL to encourage healthy eating habits | A poll |
| 39 | Share knowledge, create engagement | A recommendation for DWELL to encourage healthy eating habits | Text and pictures |
| 40 | Share knowledge, create engagement | Research information.  A recommendation for DWELL to encourage healthy eating habits | Text and question |
| 41 | Share ideas, create engagement | A recommendation for DWELL to encourage physical and mental health | Text and question |
| 42 | Thank you for participating  and end of the study questionnaire | DWELL, I COPPE and WHO-5 questionnaire + engagement questions and open questions for feedback | A text and a link to an online questionnaire |
